# The effect of vertical bracket positioning on torque and the resultant stress in the periodontal ligament—a finite element study

**DOI:** 10.1186/s40510-014-0050-0

**Published:** 2014-08-22

**Authors:** Ahmadreza Sardarian, Shahla Momeni danaei, Shoaleh Shahidi, Sahar Ghodsi Boushehri, Allahyar Geramy

**Affiliations:** Student Research Commitee, Department of Orthodontics, Orthodontics Research Center, School of Dentistry, Shiraz University of Medical Sciences, Shiraz, 71956-15878 Iran; Department of Orthodontics, Orthodontics Research Center, School of Dentistry, Shiraz University of Medical Sciences, Shiraz, 71956-15878 Iran; Department of Oral and Maxillofacial Radiology, Biomaterial Research Center, School of Dentistry, Shiraz University of Medical Sciences, Shiraz, 71956-15878 Iran; Department of Orthodontics, Dental Research Center, School of Dentistry, Tehran University of Medical Sciences, North Kargar Street, Tehran, 14399-55991 Iran

## Abstract

**Background:**

The ideal built-in tip and torque values of the straight wire appliance reduce the need for wire bending and hence reduce chair time. The vertical position of the bracket on the tooth surface can alter the torque exerted on the tooth. This is a result of the altered surface curvature observed at each vertical position. To further clarify the role of vertical bracket positioning on the applied torque and the resultant stresses in the periodontal ligament (PDL), we designed a mandibular first premolar using finite element modeling.

**Methods:**

Cone beam computed tomography of 52 patients (83 lower first premolars) was selected to be included in the study. Curvature was measured for points along the labial surface with increasing distances (0.5 mm increments) from the cusp tip by calculating the angle between tangents drawn from these points and the axis joining the cusp tip and the root apex. The mean values for each distance were calculated, and a finite element model was designed incorporating these mean values. The resultant stress and hydrostatic pressure in the PDL were calculated using finite element analysis.

**Results:**

The labial surface of the mandibular first premolar demonstrated a 26.39° change from 2.5 to 6 mm from the cusp tip. The maximum Von-Mises stress and hydrostatic pressure in the PDL were observed at the root apex for all of the bracket positions, and these values demonstrated, respectively, a change of up to 0.059 and 0.186 MPa between two successive points.

**Conclusions:**

It can be concluded that the variation in the vertical position of the bracket can have an important effect on the torque and subsequently on the stresses and pressures in the PDL.

## Background

Angle introduced the edgewise system based on a 3-dimensional tooth control obtained by engaging a rectangular wire into a bracket with a rectangular slot [1]. Andrews, taking advantage of the control offered by the edgewise system, advocated the use of the straight wire appliance (SWA) [2]. The mainstay of the SWA is the omission of tedious archwire bending by using tooth-specific brackets incorporating key attributes governing the final tooth position [3,4]. Considering that SWA brackets contain the necessary information for the desired tooth position (i.e., tip, torque, height, and rotation) [5], the only variables are tooth morphology and the position of the bracket on the surface of the tooth. While acknowledging the numerous benefits of the SWA, years of clinical practice and experience have shown that the need for archwire bending is yet to be completely eliminated [6,7]. Errors in bracket placement will lead to discrepancies in the final tooth positions [8]. Vertical bracket positioning and its effect on torque and incisor inclination have been the subject of several studies [9–12]. As much as 10° variation in torque has been observed when bracket placement differed by 1 mm [12]. Even if bracket attachment is performed perfectly, the variation in tooth morphology would render any prescription insufficient in obtaining ideal tooth positions [8].

Root resorption, a sequel of orthodontic treatment, has been linked to the amount of torque applied to a tooth [13–15]. Torqueing of a tooth displaces the root apex horizontally which has been shown to induce root resorption [16]. Studies evaluating the relationship between torqueing force and root resorption arrived at the same conclusion, that is, higher torque magnitudes result in elevated resorption [13,15].

In the present study, we chose to assess vertical bracket positioning on the mandibular first premolar as these teeth have been shown to posses the highest values for labial surface curvature (excluding mandibular molars) [12]. Therefore, slight errors in bracket placement could lead to a more significant change compared to other teeth (incisors, canines), which demonstrates a flatter labial surface. Another reason for the evaluation of mandibular premolars is that often, there is an increased overbite in this area, and there is a tendency to bond the bracket further gingivally in order to prevent contact with the opposing teeth. This is likely to result in undesired torque forces due to the labial surface curvature of the premolars.

We aimed to design a finite element model of a mandibular first premolar with a labial surface incorporating the mean curvature derived from a sample of cone beam computed tomography (CBCT) images. To our knowledge, this method has not been reported previously. Using this model, we will calculate the variable torque due to vertical bracket positioning and the resultant stresses in the periodontal ligament (PDL).

## Methods

### Sample

A total of 52 CBCTs containing both jaws obtained with a New Tom VGi (Quantitative Radiology, Verona, Italy) at 3.05 mA, 110 kV, and an exposure time of 3.6 s with a voxel dimension of 125 μm were selected randomly for measurement of labial surface curvature. The CBCT images had been obtained for nonorthodontic reasons. The inclusion criteria for the CBCT images were radiographies obtained from healthy individuals who were 15 to 30 years old, who did not suffer from any syndrome known to cause altered tooth morphology, and who had a normal class I occlusion. Furthermore, the CBCT views were discarded if the mandibular first premolar was affected by caries, had restorations, was subjected to severe attrition or fractured, and if it possessed an abnormal or dilacerated root. After the application of the exclusion criteria, 83 premolars were left to be included in the study.

### Radiographic measurements

**Figure 1:**
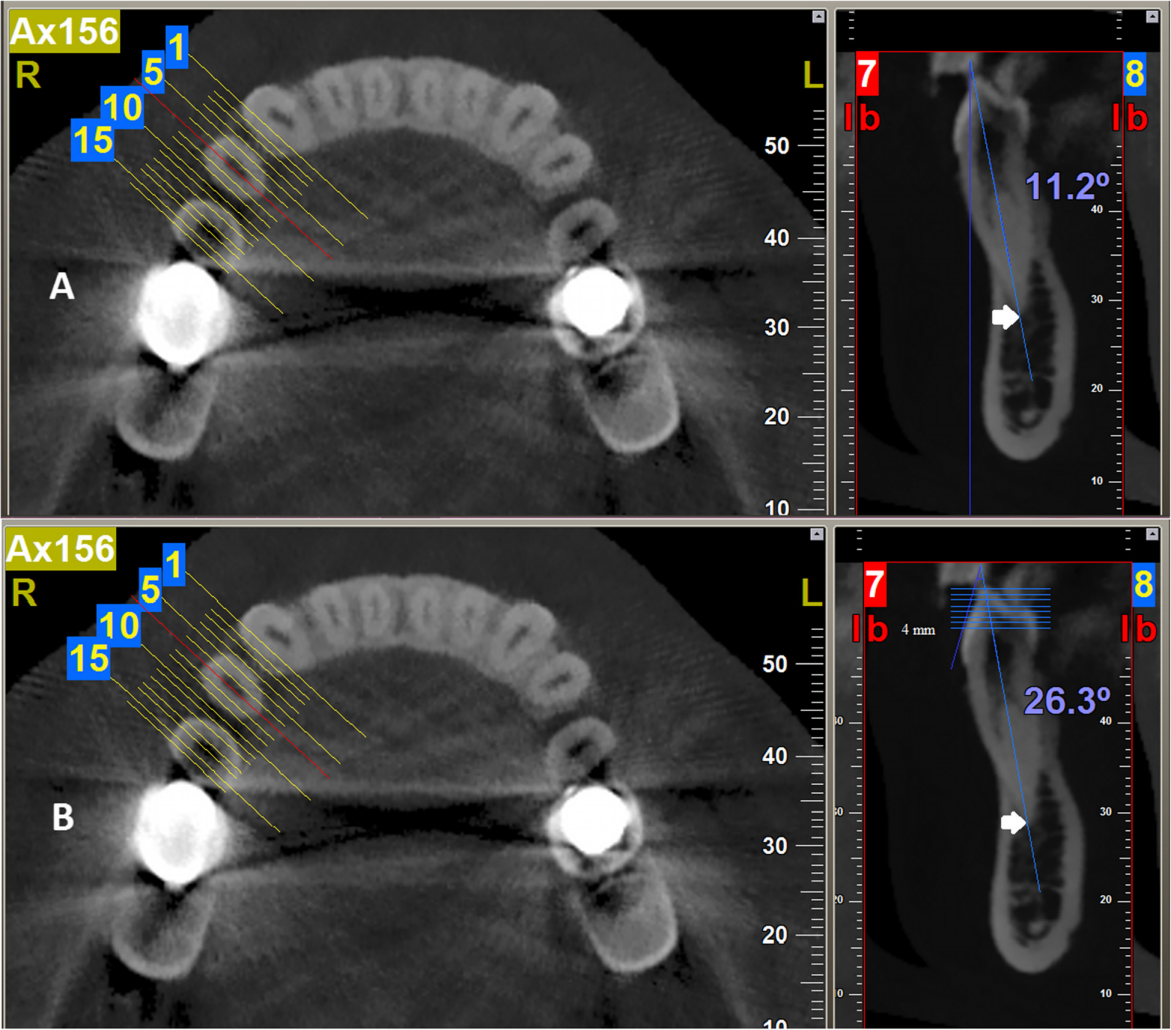
**Measurements of the angles. (A)** Measurement of the angle between the axis connecting the apex and the cusp tip of the mandibular first premolar and the true vertical. As can be observed, sections were made buccolingually, and the section pertaining to the mesiodistal width of the tooth was used. **(B)** Measurement of the labial surface curvature. The arrow indicates the axis connecting the cusp tip to the apex. The horizontal lines are 0.5 mm apart. The measurement of the angle is performed between the axis of the tooth and tangents drawn at the intersection of the horizontal lines and the labial surface.

A section was made passing through the mesio-distal center of the crown. On this section, tangents were constructed from points on the labial surface of the tooth with increasing vertical distances (0.5 mm increments) from the cusp tip. The angle between each tangent and the axis connecting the cusp tip and the apex and also the angle between the mentioned axis and the true vertical were calculated (Figure 1). All the measurements were made using NNT viewer version 2.21 (Quantitative Radiology, Verona, Italy). The measurements were performed twice by a board-certified oromaxillofacial radiologist at a 3-month interval.

### Statistical analysis

Intra-observer reliability was determined using the intra-class correlation coefficient (ICC). Mean curvature for each point on the labial surface was calculated. All the statistical analyses were performed using the Statistical Package for Social Sciences for Windows 11.0 (SPSS Inc., Chicago, IL, USA).

### Finite element modeling

**Figure 2:**
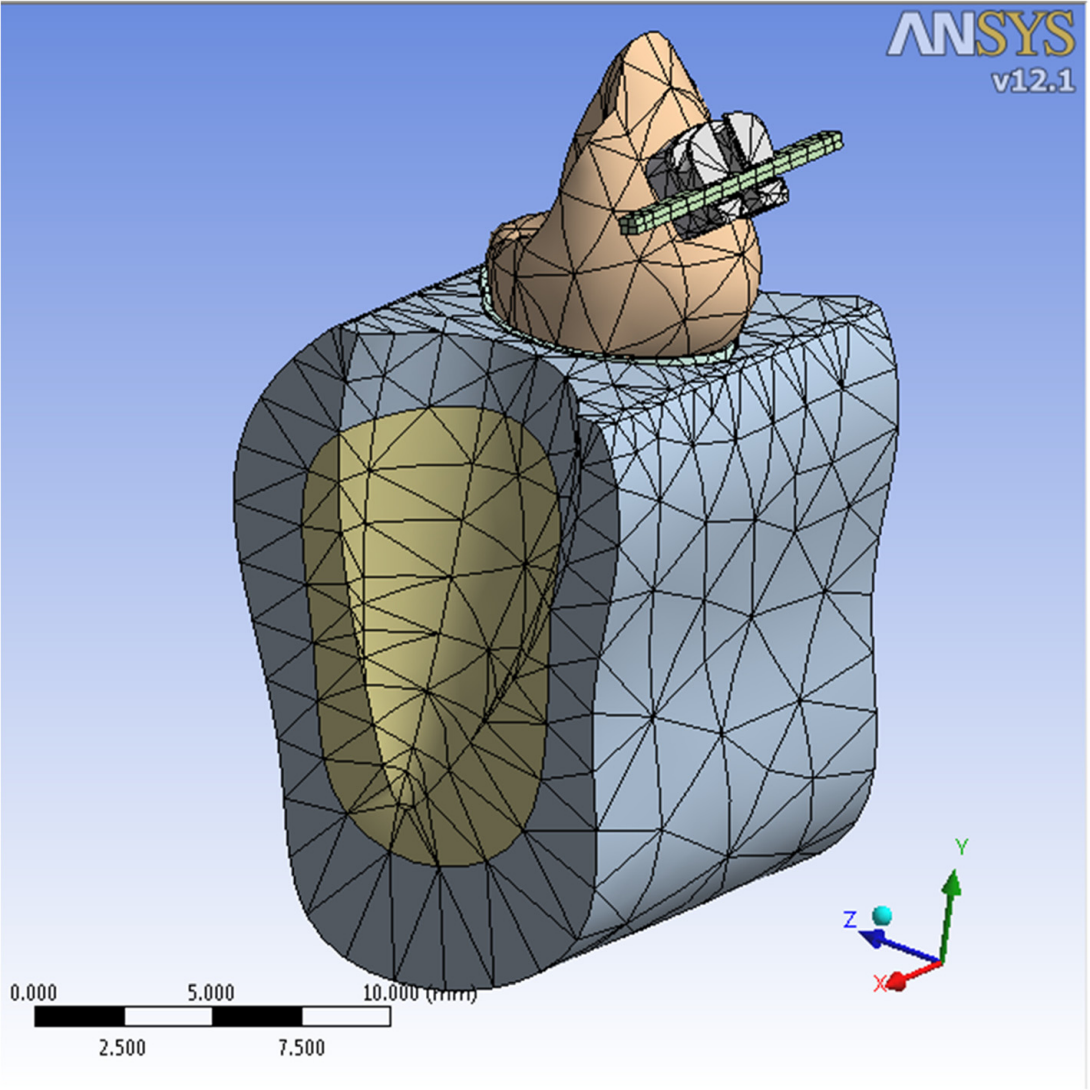
**The finite element model of the first mandibular premolar.**

Ten 3-dimensional finite element models of the mandible were designed in SolidWorks 2006 (300 Baker Ave. Concord, MA, USA). The models contained a mandibular first premolar, its PDL, and the surrounding cortical and cancellous bones (Figure 2). The labial surface of the tooth was modeled in a way to represent the study sample. This was accomplished by aligning the axis connecting the cusp tip and the root apex vertically and modeling a curve best fitting the obtained mean values of surface curvature. Other dimensions of the tooth including crown height (8.5 mm), root length (14 mm), buccolingual width (7.5 mm), and mesiodistal width of the crown (7 mm) were designed in concordance to Wheeler's text on dental anatomy [17]. The PDL was designed with a uniform thickness of 0.25 mm. An 0.022-in. stainless steel bracket was designed, incorporating Roth's prescription for torque of the mandibular first premolar (−17°). The bracket was ‘attached’ to the tooth surface, and composite was added to fill the gaps between the bracket and the tooth. The bracket's vertical position initiated at 1.5 mm (distance between the center of the slot and the cusp tip) which was the most occlusal distance that provided a good fit between the bracket base and the tooth surface. The bracket was then displaced 4.5 mm gingivally in 0.5-mm increments between subsequent models. A 19 × 25 stainless steel wire was engaged into the bracket, and the distance from the corners of the wire to the horizontal plane was calculated in the designing software. This measurement was later used for statistical analysis in the ANSYS Workbench 11.0 (South Pointe, 275 Technology Drive, Cononsburg PA, USA). The elastic modulus and Poisson's ratio were defined for the different entities of the model based on the previous studies [14,18] (Table 1). The number of elements (10-node-quadratic tetrahedron) for the tooth and PDL was 16,386 and 18,156, and the number of nodes was 33,345 and 36,318, respectively (Figure 2). As the boundary condition, all nodes at the base of each model were restricted from displacements in all directions. Geometric nonlinearities in the PDL were allowed following the application of force. Each analysis consisted of eight displacements: four in the left extreme and four in the opposite side extreme of the wire. The displacements applied were calculated trigonometrically using SolidWorks. The rectangular wire was placed in an angulated position when inserted into the bracket in complete contact with the walls of the slot. Then the displacements were applied to rotate the wire to a horizontal position. This method of calculation takes into account the play that exists between the bracket and wire. The vertical position of the bracket and wire was matched so that no vertical activation of the wire occurred. This setting could relate to the clinical circumstances because rectangular stainless steel wires are applied after leveling has been obtained using more resilient wires initially. The Von-Mises stress and also the hydrostatic pressure (defined as S_1_ + S_2_ + S_3_/3) in the PDL of the tooth was calculated for the various bracket positions.

**Table 1:** **Parameters for the mechanical properties of tooth, spongy bone, cortical bone, PDL, and stainless steel**

	**Young's modulus (MPa)**	**Poisson's ratio**
Enamel	84,100	0.33
Dentin	18,600	0.30
Alveolar bone	490	0.30
Cortical bone	14,700	0.30
PDL	0.1	0.45
Stainless steel	200,000	0.30
Composite	2,140	0.31

## Results

### Buccal surface curvature

**Figure 3:**
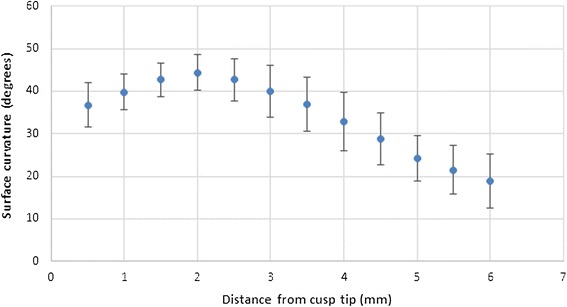
**Labial surface curvature at variable distances from the cusp tip (± SD).**

The ICC was used to determine the agreement between the two measurements made by the OMFR. The results demonstrated an excellent agreement of 0.92 (95% confidence interval, 0.89–0.95). The average buccal surface curvature at variable distances (0.5 to 6 mm) from the incisal edge is presented in Figure 3. The values correspond to the angle between the tangent to the buccal surface at each distance and the long axis of the tooth (Table 2).

**Table 2:** **Buccal surface curvature at variable distances from the cusp tip of first mandibular premolars**

**Distance (mm)**	**Maximum**	**Minimum**	**Maximum-Minimum**	**Mean (SD)**	**∆ Mean**
0.5	47.1	26.1	21	36.73 (5.2)	
1	45.5	30.1	15.4	39.81 (4.2)	−3.08
1.5	48	34.7	13.3	42.67 (4)	−2.87
2	49.3	37.9	11.4	44.3 (4.2)	−1.63
2.5	49.6	29	20.6	42.7 (5)	1.62
3	49.1	29	20.1	39.99 (6.1)	2.69
3.5	46.7	26.4	20.3	36.91 (6.4)	3.09
4	48.9	25.6	23.3	32.92 (6.8)	3.99
4.5	44.5	23.8	20.7	28.8 (6.2)	4.12
5	38.1	19.7	18.4	24.2 (5.4)	4.6
5.5	31.5	15.8	17.7	21.51 (5.7)	2.69
6	29.7	14.1	15.6	18.91 (6.3)	2.6

∆ Mean, the difference between the mean surface curvatures of two subsequent vertical distances.

The buccal surface curvature had the most variability at 4 mm from the incisal edge with the maximum and minimum values measured at 48.9° and 25.6°, respectively. Between 2.5 and 6 mm, the buccal surface curvature decreases steadily, although between 4 and 4.5 mm and also between 4.5 and 5 mm, a larger change was observed. A total difference of 26.39° exists between 2 and 6 mm from the incisal edge (Figure 3). The mean inclination of the axis connecting the apex and the cusp tip relative to the true vertical line was 12.1°.

### Stress and strain at the composite interface

**Figure 4:**
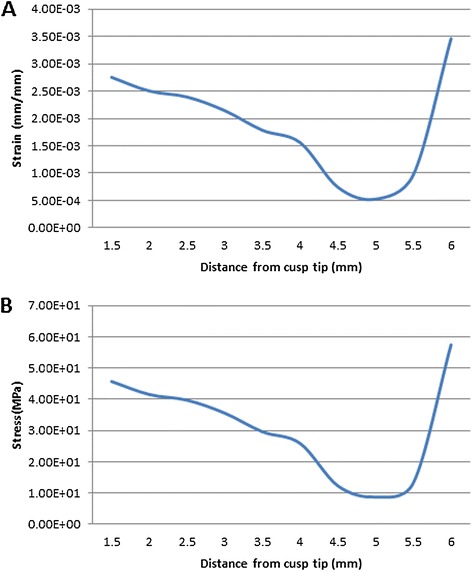
**Strain and Von-Mises stress in the composite interface.** Strain **(A)** and Von-Mises stress **(B)** in the composite interface between the bracket and the labial surface of the mandibular second premolar.

Following the virtual engagement of the wire into the slot of the bracket the bracket, the stress and strain occurring in the composite interface were calculated. Figure 4 demonstrates these values for the variable distances of bracket placement from the cusp tip. The stress reduces gradually as the distance increases from the most occlusal position of the bracket. A minimum stress of 8.7 MPa was recorded at 5 mm. Further increase in the distance of the bracket from the cusp tip resulted in increasing levels of stress, reaching a maximum of 57.5 MPa at 6 mm. The strain levels demonstrated a similar pattern to those observed for stress. The minimum strain was also observed at 5 mm (0.00074), while the maximum strain occurred at 6 mm (0.0035). The torque exerted on the tooth following the engagement of the wire was calculated (Table 3). The lowest torque was at 5 mm from the cusp tip. Displacing the bracket 1 mm occlusally or gingivally alters the torque applied to the tooth by 23 and 71 Nmm, respectively.

**Table 3:** **Torque exerted on the tooth for brackets at different vertical distances from the cusp tip**

**Distance (mm)**	**Torque (Nmm)** ^**a**^	**∆ Torque**
1.5	−30.186	
2	−28.557	1.629
2.5	−27.11	1.447
3	−26.312	0.798
3.5	−22.879	3.433
4	−19.887	2.992
4.5	−8.559	11.328
5	4.588	3.971
5.5	29.306	−24.718
6	42.937	−28.631

^a^Positive values demonstrate lingual crown torque. ∆ Torque, difference of torque between two subsequent bracket positions.

### Root displacement

**Figure 5:**
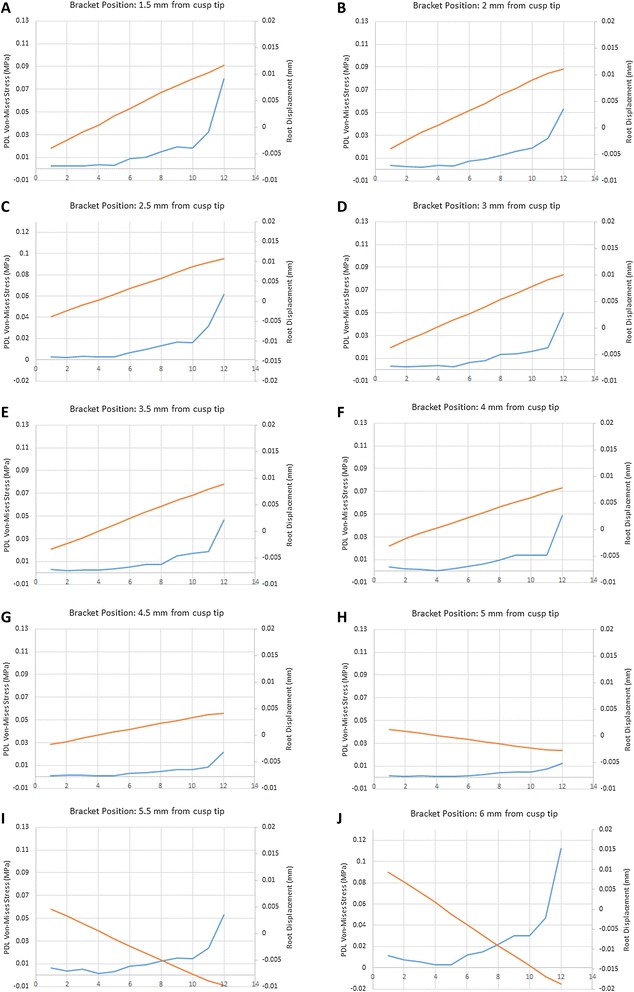
**Root displacement and Von-Mises stress for each bracket position.** Root displacement and Von-Mises stress for each bracket position ranging from **(A to J)** 1.5 to 6 mm from the cusp tip. The displacement and stress are measured along 12 points on the root from the CEJ to the apex. The red and blue lines pertain to root displacement and Von-Mises stress respectively.

The root displacement of the mandibular first molar subjected to torque was measured at selected points (Figure 5). Negative values pertain to movements in the buccal direction. Minimum displacement is observed at a similar distance (point 3 on the root, about 4 mm from the CEJ) for all of the bracket positions which gives assurance about the soundness of the model and the obtained results.

### Stress in the PDL

The Von-Mises stress in the PDL surface adjacent to the roots was calculated for the various bracket distances (Figure 5). The highest stress was observed at the root apex regardless of the level of the bracket. The stress in the PDL at the level of the root apex was largest when the bracket was placed at 6 mm followed by 1.5 and 2 mm (Table 4).

**Table 4:** **Maximum Von-Mises stress and maximum hydrostatic pressure calculated for various bracket distances from the cusp tip**

**Distance (mm)**	**MVS (MPa)**	**∆ MVS**	**MHP (MPa)**	**∆ MHP**
1.5	0.0793		0.2749	
2	0.0633	0.016	0.259	0.0159
2.5	0.0617	0.0016	0.253	0.006
3	0.0496	0.0121	0.251	0.002
3.5	0.0467	0.0029	0.238	0.013
4	0.0445	0.0022	0.186	0.052
4.5	0.0214	0.0231	0.105	0.081
5	0.0124	0.009	0.058	0.047
5.5	0.053	−0.0406	0.201	−0.143
6	0.112	−0.059	0.387	−0.186

MVS, maximum Von-Mises stress; ∆ MVS, difference between MVP of two subsequent bracket positions; MHP, maximum hydrostatic pressure; ∆ MHP, difference between MHP of two subsequent bracket positions.

Similar to Von-Mises stress, hydrostatic pressure was also maximum at the tooth apex in the direction of root displacement. The highest pressure was measured when the bracket was placed at 6 mm and the lowest at 5. The difference in the maximum hydrostatic pressure between subsequent bracket distances was calculated. The highest difference was between 5.5 and 6 followed by 5 and 5.5 mm from the cusp tip (Table 4).

## Discussion

During the course of this study, a mandibular first premolar was designed, incorporating a labial surface curvature in concordance with a population of individuals with normal occlusion. Preadjusted edgewise brackets were then placed on the mentioned labial surface at variable distances from the cusp tip, and a full size archwire was virtually engaged into the bracket and the resultant torque, root displacement, and PDL stress were calculated.

The labial surface curvature was measured by calculating the angle between tangents at various vertical distances along this surface and a line extending from the cusp tip to the apex of the first mandibular premolar. This method is similar to the method advocated by Van Loenon et al. who measured the labial surface curvature of the maxillary central incisor and canine [11]. In their study they obtained a proximal radiograph from the teeth and digitized their images for measuring the angle between tangents to the labial surface and the long axis of the crown. We opted to use CBCT for the purpose of obtaining the proximal view and the measurements were done on the NNT viewer software. Using CBCT and direct measurement reduces errors regarding magnification, tooth orientation, and digitization of the images. Another method for the determination of labial surface curvature, which has been described by several authors, uses a device that engages in a standard edgewise bracket bonded on the tooth surface [19,20]. This method also has some room for error, as the trimming of the cast should be accurate to ensure parallelism of the occlusal plane and the base of the cast. Furthermore, as the horizontal plane is used as reference for determination of the curvature, another factor, namely, the inclination of the tooth in the jaw also influences the results. Using a reference line constructed by anatomic landmarks on the tooth itself reduces extrinsically introduced errors. We measured the inclination of the apex-cusp tip axis with the true vertical, and because it was observed that the axis is inclined buccally by 12.1°, by subtracting this value from the labial surface curvature values, we can arrive at numbers similar to those present in the literature [19–21]. The ICC value for intraobserver agreement in the present study was 0.92 (95% CI, 0.89–0.95), which demonstrates excellent agreement between the two measurements. This high intraobserver agreement has been previously reported by other authors investigating the reliability and repeatability of measurements based on CBCT images [22,23].

The displacement values obtained after the wire was virtually engaged in to the bracket slot represent the movement of the root of the tooth in the PDL. Looking at the displacement chart, the lines corresponding to the different vertical positions of the brackets all seem to cross at about 4 mm from the cementoenamel junction (CEJ), which is the location corresponding to the center of resistance in our model tooth. The Von-Mises stress values confirm the displacement data so that the highest stress is observed in the areas demonstrating the largest amounts of displacement. Again, the lowest stress values were measured at 4 mm from the CEJ. Analyzing the data, we can deduce that the least amount of stress at the root apex was measured when the bracket was bonded 5 mm from the cusp tip. By displacing the bracket gingivally from 5 to 6 mm, a ninefold increase occurs in the stress level measured at the root apex, while moving the bracket occlusally to 4 mm resulted in four times as much stress on the opposite side of the root. These results shine light on the importance of proper bracket positioning when using the straightwire appliance. In this study we used the Von-Mises stress defined as $\frac{1}{\sqrt{2}}{\left[{\left( S1- S2\right)}^2+{\left( S2- S2\right)}^2{\left( S3- S1\right)}^2\right]}^{0.5}$, which has been used in the previous studies to assess the stress environment of the PDL [24–26]. Subjecting a tooth to torque could have potential root resorbing side effects [13,14]. In a study by Hohmann et al., it was observed that torque applied to a tooth causes root resorption and that such resorption could be predicted using finite element analysis. In their study they opted to use hydrostatic pressure and concluded that when hydrostatic pressure surpassed 0.0047 MPa, the value corresponding to capillary blood pressure, root resorption occurs. We therefore calculated the hydrostatic pressure defined as S1 + S2 + S3/3, for the different vertical positions of the brackets to assess the probability of root resorption. The hydrostatic pressure values obtained from our study are much greater than those observed by Hohmann et al. when they subjected the tooth to 3 and 6 Nmm of torque. This can be explained by the fact that wire engagement in our study resulted in higher torque forces, the minimum of which was 9.59 Nmm with the bracket positioned 5 mm from the cusp tip. It has been claimed that torquing force of 5–20 Nmm is acceptable clinically [27], and based on the results of the present study, bonding the bracket just 1 mm more gingivally than the 5 mm point could result in torquing forces exceeding the mentioned amount. As demonstrated by Hohmann et al. and several other authors, significantly more root resorption occurs by increasing the torque exerted on the tooth [13–15].

Finally, considering the stress levels in the composite interface between the bracket and the tooth, they appear to change to critically large values by only moving the bracket 1 mm gingival or occlusal from the location where minimum stress levels were calculated (5 mm from the cusp tip). Shear bond strength values for orthodontic composite resins have been reported in literature, which consist of a wide array of values ranging from 5.9 to 25.5 MPa for metallic brackets [28,29]. While the numbers reported in the present study are Von-Mises stress values and therefore cannot be directly related to the shear bond strength values, they can demonstrate the possible effect of incorrect bracket positioning on increased debonding rate of brackets. There is room for future research regarding this hypothesis in laboratory and clinical settings.

The limitation of this study as with other finite element studies of tooth movement was the assumption that the PDL possesses isotropic and elastic behaviors. Also, the virtual mechanical properties of the material differ from their actual properties. Furthermore, in the present study the neighboring teeth were not taken into account for the analysis, and it was assumed that brackets bonded on them were at the same level of the modeled tooth. Although this assumption can be justified by the fact that stainless steel archwires modeled in this study are usually inserted in the clinic once NiTi wires level the brackets. It is obvious that the position of the bracket on the adjacent teeth will have a significant effect on the results. Also, variations in the torque resulting from altered vertical bracket positions could negatively affect root resorption and debonding rate of orthodontic brackets. We suggest that future studies address the mentioned points and also analyze different wire sizes and archwire material.

## Conclusions

We can conclude that preadjusted brackets can produce a variable amount of torque depending on the vertical positioning of the bracket and its relation to tooth morphology. We have also quantified the magnitude of the alterations in the stress felt by the PDL resulting from the changes in vertical bracket positioning.
